# Personal Networks and Cervical Cancer Screening among Black Immigrant Women

**DOI:** 10.1007/s40615-024-02231-6

**Published:** 2024-11-11

**Authors:** Leslie E. Cofie, Olivia Whitt, Nikhil Bhagat

**Affiliations:** 1 Department of Health Education and Promotion, East Carolina University, 3016 Belk Building, Mailstop 529, Greenville, NC 27858 USA; 2 School of Public Health and Tropical Medicine, Tulane University, New Orleans, LA USA

**Keywords:** Black immigrants, Social networks, Papanicolaou screening, Cancer prevention

## Abstract

**Background:**

Prior research has linked personal network characteristics with cancer screening uptake including Papanicolaou (Pap) screening, but less is known about the experiences of Black immigrant women (BIW) in the USA. We examined the relationship between network characteristics and Pap screening among BIW and explored how their network members influence their cancer related knowledge and prevention behaviors.

**Methods:**

A mixed methods study of BIW, aged 21–65 years, in southeastern US included a cross-sectional survey (*N* = 204) and in-depth individual interviews (*N* = 13). We examined whether high-social connectedness, contact frequency, and social support were associated with Pap screening, using multivariable logistic regression models. Thematic analysis further assessed the roles of personal network factors on BIW’s cancer preventive behaviors.

**Results:**

Pap screening was more likely among BIW with high- versus low-social connectedness (OR: 2.68, CI: 1.12, 6.46). However, the impact of high-social connectedness was attenuated, after adjusting for demographic factors and health insurance. Our qualitative findings revealed that both BIW and their personal networks had limited knowledge on cancer and related prevention measures. Close network members, particularly mother-figures, provided support for BIW’s care seeking efforts, including cancer screening, although some participants mentioned a lack of screening support.

**Conclusion:**

These findings suggest that Black immigrant communities may benefit from tailored cancer prevention interventions among close network members, to improve knowledge and support for cancer control behaviors.

## Introduction

The US foreign-born population was approximately 45.3 million in 2021, over a fourfold increase since 1970 [1]. Many foreign-born individuals in the USA are from various world regions, particularly low- and middle-income countries (LMICs). For example, in 2020, foreign-born individuals in the USA included those from Africa (6%), Asia (35%), and the Americas (52%) [2]. These LMIC regions are known to have elevated cancer risks and sub-Saharan Africa in particular has the highest cervical cancer incidence and mortality[3, 4]. Black immigrants are one of the fastest growing foreign-born populations in the USA; they presently makeup nearly 10% of the USA. Black population and are projected to reach 17% by 2060 [5]. National cancer surveillance programs and prior research have portrayed US Blacks as homogeneous, thus overlooking differences in the cancer related experiences of various Black subgroups. Recent works have, however, provided evidence of heterogeneity in cancer incidence, survival, and risk factors among different subpopulations of Blacks in the USA [6–8].

Cancer remains the second leading cause of death globally, and a major cause of morbidity across LMIC regions. This is in-part due to rapid globalization in LMICs, which has contributed to spread of cancer risk factors such as environmental and occupational pollutants, tobacco use, noncommunicable diseases (e.g., diabetes and obesity), physical inactivity, and unhealthy diets [9–11]. Before migrating to the USA, foreign-born individuals are susceptible to certain infection-related cancers, e.g., liver, stomach, and cervical cancers [7, 12]. Despite these risk exposures, immigrants from LMIC regions likely have limited cancer knowledge and access to prevention, early detection, and treatment services in their home countries [13]. Upon migrating to the USA, they further experience challenges navigating the US healthcare system including accessing cancer prevention and early detection services [14]. The challenges experienced by immigrants, especially those from LMIC, may be attributed to factors including low English proficiency, limited knowledge of the US healthcare system, lack of health insurance, uncertainty about immigration status, and state and federal policies related to healthcare access for immigrants [15, 16].

Secondary cancer prevention including early screenings—e.g., for Papanicolaou (Pap) test and mammogram—are recommended as effective clinical preventive measures for reducing disease incidence and mortality [17, 18]. Yet, screening uptake among foreign-born individuals is lower than those born in the USA [19–22]. Previous research suggests that foreign-born women from various world regions (e.g., South America, Caribbean, Southeast Asia Central Asia (20.4%), Middle East, and Africa) were significantly less likely to report cervical cancer screening uptake than US-born women [23–25]. Among US immigrants of African descent, cervical cancer screening rates were markedly low, and significantly lower than the screening rates of US born women [23, 26, 27]. Specifically, low rates of cervical cancer screening have been observed among Caribbean- and African-born Black women [26, 28]. Also, recent evidence suggests that Black immigrant women (BIW) were seven times less likely to report up-to-date cervical cancer screening than African American women in the USA [27].

Prior research on factors associated with cancer screening among immigrants can be understood within the context of the socioecological framework [28–30]. For instance, individual determinants of screening include cancer-related knowledge and personal attitudes or beliefs about screening (e.g., fear, embarrassment or modesty when seeking screening) [31, 32]. Interpersonal level factors associated with screening include cultural and fatalistic beliefs about cancer risks, and spousal consent for screening [33]. Organizational and community determinants of screening may include physician recommendation to get screened, availability of culturally competent care such language interpretation services, and broader structural racism within the healthcare sector [34, 35]. Additionally, practical challenges to receipt of screening may include finding childcare, scheduling appointments, and working long hours [32, 34]. The sociocultural context of immigrants in terms of their social relationships is an interpersonal level determinant that has been less examined, despite being crucial to comprehending how screening behaviors may be improved among this group.

The present study focuses on Black immigrant women’s social relationships, which is an interpersonal level factor within the socioecological framework. The social relationships of immigrants make up their social networks. Social networks is described as one’s relationship with network members (e.g., relatives, friends, neighbors, co-workers) and this relationship is considered as having both a structure and serving various functions [36]. One’s social network structure may be composed of different types of network members with whom one has varying levels of interactions. The network members may function in terms of influencing one’s behaviors and providing various types of social support [36]. Social support is defined as the extent to which individuals receive aid (e.g., material resources, advice, and emotional support) from network members when they need it [37].

Prior work has linked network characteristics with health behaviors including cancer screenings like Pap tests [38–41]. Lager social networks and higher social integration or connectedness (based on the index of network size, frequency of contact with network members and church membership) have been positively associated with screenings for breast cancer [42, 43], colorectal cancer [44], and cervical cancer [38, 45], among US adults. Research also suggests that having social support from close network members is associated with cancer preventive behaviors including mammography and breast and colorectal screening adherence [46, 47]. There is evidence of this relationship among various race/ethnic minority groups including African Americans, Hispanics [38, 48], and Asians [49]. Conversely, examination of the relationship between network characteristics and social support with cancer screening among foreign-born individuals is sparce, especially among Black immigrants. To date, Adegboyega found that positive social interactions and affectionate support was associated with Pap testing among sub-Saharan African immigrant women.

Foreign-born groups have unique personal networks and often emigrate because of social and family ties in their host country [50]. Like all foreign-born groups, the characteristics and roles of Black immigrant women’s personal networks can change over time. Thus, it is critical to understand how women’s personal networks influence cancer preventive behaviors, as well as ways they may be leveraged to increase cervical cancer screening adherence. We focus on Black immigrant women because they have distinct cultural backgrounds and experiences from their US-born counterparts, which impact their cervical cancer risks. For example, due to high rates of cervical cancer incidence and mortality in sub-Saharan Africa, immigrant women from the continent have an increased risk of the disease [3]. Unlike the USA, cervical cancer screening access and use is limited and not common practice in regions like Africa [51–53]. Disaggregating Black immigrants from the larger US Black population is crucial for gaining a nuanced understanding how an interpersonal level factor like personal networks influences their cervical cancer screening uptake upon migrating the USA. It also adds to the limited discourse on cancer health disparities experienced by various ethnic Blacks in the USA.

To that end, we examined the association between social network characteristics and Pap test uptake among Black immigrant women using both quantitative and qualitative methods. Specifically, we aimed to quantitively assess whether having a strong social connection with their social (i.e., personal) networks and receiving social support from them was related to an increased uptake in cervical cancer screening (Pap test). We also qualitatively explored how the personal networks of the women influenced their health decisions and behaviors including their cancer related knowledge and prevention behaviors. The qualitative examination of the women’s experiences was intended to provide an in-depth understanding of how network members contribute to the Black immigrant women’s cancer screening behaviors.

## Methods

### Study Design

A Black Immigrant Women Study was conducted between 2020 and 2021 using a mixed methods approach. The study’s objective was to examine the role of personal network factors on cancer screening behaviors of Black immigrant women in southeastern United States. Quantitative and qualitative data were separately collected and analyzed to enable qualitative interpretation of the statistical findings [54]. The quantitative data were derived from a cross-sectional survey administered to a convenience sample of women. The qualitative data was from in-depth individual interviews with a sub-sample of those women. By examining personal network characteristics and Pap test uptake through both quantitative surveys and qualitative in-depth interviews, we enhance the credibility of our findings through methodological triangulation. We integrated the findings from both approaches to gain a comprehensive understanding of how Black immigrant women’s interactions with their personal network relate to their Pap test uptake. This study was exempt from ethics review by East Carolina University (UMCIRB 19–000571).

### Participant Recruitment and Sample

#### Quantitative Sample

We initially planned to recruit women in North Carolina (NC) cities with sizeable foreign-born populations including Black immigrants. The COVID-19 pandemic caused recruitment challenges, so we pivoted to working with the company Qualtrics to help recruit study participants. Qualtrics used various national panel sources to recruit individuals that fit the inclusion criteria of a study [55]. Women in NC were originally targeted, but due to the limited number of responses (~ 30 women), we expanded our recruitment to women in South Carolina, Georgia, Alabama, Virginia, and Maryland. We developed an online-based survey, which Qualtrics administered to its participant pool, that met our inclusion criteria. The inclusion criteria were (1) Black women born outside of the USA; (2) have lived in the country for at least a year to ensure that they have established residency; and (3) were between the ages of 21–65 years, which is the recommended age range for Papanicolaou (Pap) testing among women in the USA [17]. Data quality check in Qualtrics included checking for responses to specific questions to ensure accuracy, checking for duration of time spent on the survey compared with the median [55]. The administered survey yielded 213 online survey responses.

Later, as the pandemic restrictions were eased, we collaborated with a community leader in the Black immigrant community in NC, who leveraged her networks to help recruit 90 additional women who took the survey. Through word of mouth, she recruited women from her local network of Black immigrant-focused civil societies and community-based organizations. Interested study participants were connected with the data collector who in turn provided them with more information about the study. Women who met the inclusion criteria were provided access to the survey. The survey response data from this community was combined with the survey data from the Qualtrics panel. The analytic sample for this study was 204 after excluding women with missing information on all key variables of interest.

#### Qualitative Sample

Additionally, the community leader helped recruit a subsample (*n* = 13) of the 90 women surveyed in NC to participate in individual interviews. Utilizing community members as recruiters is a recommended strategy to find hard-to-reach or minority communities and to build trust and increase inclusion in clinical and public health research [56, 57]. The community leader facilitated access to women in the community and connected the first author and data collector with Black immigrant community organizations. Thus, creating opportunities for us to establish rapport with women in the community.

### Data Collection

#### Quantitative Measures

A health survey instrument developed and administered to the women included questions on social network characteristics, cancer screening behaviors, health access factors, and demographic questions. The *outcome measure*, cervical cancer screening, was based on up-to date screening as defined by the US Preventive Services Task Force (USPSTF); i.e., a recommendation for women ages 21 − 65 of average risk to get a Pap test every three years [17]. The survey question was derived from the was National Health Interview Survey [58]. Women were asked how long it has been since their last Pap test. The response categories “a year ago or less,” “more than 1 year but not more than 2 years,” or “more than 2 years but not more than 3 years,” were coded as “yes”; the rest were coded as “no.”

*Key independent measures* were derived from the Berkman-Syme Social Network Index [59, 60], a widely adopted valid and reliable quantitative measure of social relationships [61]. This index includes four types of social connections: marital/partnership status (married/with partner vs. not), sociability (which examines number and frequency of contact with close relatives, and close friends), church group membership (yes vs. no), and membership in community organizations (yes vs. no). Details on the network index are described elsewhere [59, 62, 63]. Social connectedness was measured as a composite score of the four types of connections and categorized into low, medium, and high levels. Sociability was also categorized into 3 levels based on number and frequency of intimate contact (low, medium, and high).

*Social support* was assessed using a composite score of five items from the social network index related to the availability of someone to give advice show love and affection, provide emotional support, and trust and confide in. The response options (none, 1 or 2, 3 to 5, 6 to 9, 10 or more) were categorized into high, medium, and low. We developed an additional personal network-related question to assess screening support, “If you need to get cancer screening (e.g., cervical and breast cancer screening), do you feel that you have some relatives and close friends that you can rely on to help get you there or tell you where to go?” We coded the response categories “definitely have” as high, “probable have” and “might have” as medium, and “probably don’t have” as low screening support.

*Control measures* were adopted from the National Health Interview Survey comprised of health access, length of US residency, and demographic factors [58]. Health access factors included having insurance (yes vs. no) and perceived health status (categorized as excellent/very good, good, or fair/poor). Length of US residence was a measure of how many years the women have lived in the USA; their responses were grouped into < 5, 6–15, and > 15 years). Demographic measures were age, marital status, education, and household income. These measures are important factors that have previously been associated with cervical cancer screening [64].

#### Qualitative Assessment

An interview guide was developed for the individual interviews The questions included (1) what are women’s perceptions about cancer control and prevention, (2) what were their previous experiences with cancer control in their home country, (3) who are members of their social networks and what is their level of interactions with them, and (4) what are the roles of the network members in promoting health related knowledge, access, and support for preventive behaviors including cancer screening? This guide was developed with input from the research team and the extant literature on the experience immigrant women’s social network experiences. A trained female data collector conducted and audio-recorded phone interviews with the women via WhatsApp. The data collector was trained in qualitative research methodology, ethical considerations for working with vulnerable populations, and skills for establishing rapport with minority immigrants. The sample size was adequate to reach data saturation (*n* = 13) [65]. Participants’ ages ranged from 33 to 65 years old. They emigrated from Africa as adults, and their length of US residency ranged from 1 to 21 years. Each woman interviewed was verbally consented before participating in the study and received a $20 gift card after completing the interview.

### Data Analysis

**Quantitative analyses** were conducted in SAS version 9.34 (SAS Institute, Cary NC). Using chi-square test and *t*-test, we examined whether social connectedness, contact frequency, social support and screening help and advice support were associated with up-to-date pap screening. Unadjusted logistic regression models were used to test the association between the personal network measures and pap screening. Prior literature has indicated that insurance status is significantly associated with Pap test uptake among women including Black immigrants [34, 66]. We assessed the interaction between the network factors and insurance, but the effects were insignificant. Subsequently, an adjusted multivariable regression model was ran using stepwise regression with forward selection. The control measures for the final regression model included insurance, health status, age, marital status, and income. The regression analyses were two-tailed (*p* < 0.05) with a 95% confidence interval.

*Qualitative analysis* was conducted using the ATLAS.ti software (versionver7.0, Scientific Software Development GmbH, Eden Prairie, MN). First, NB developed narrative summaries of women’s experiences with the personal networks in their health seeking behaviors. We (LC, NB, and OW) then begun initial close readings of the interview transcripts. A preliminary codebook was developed based on deductive codes from the interview guides and inductive codes from the transcripts. Specifically, we met to discuss emerging ideas from the transcripts, narrative summaries and interview memos to generate the inductive codes; deductive codes were derived from the interview guide questions. NB and OW independently applied the codes to the transcripts and suggested new codes as new ideas emerged during analysis. NB and OW met regularly with LC for coding quality checks and to ensure coding reliability. Any differences in coding and code creation were discussed until a consensus was reached. If a new inductive code was added to the codebook, NB and OW re-reviewed all interviews to fully capture the phenomena. This process of consensus and multiple passes allowed for investigator triangulation and reducing the impact of individual biases. The two coders later developed code output summaries of parent nodes and included representative quotes. They then worked with LC to analyze the output and narrative summaries by creating analytic matrices [67, 68], which identified salient themes related to women’s perspectives of how their personal networks influence their health decisions and experiences, particularly related to cancer prevention.

### Investigator Reflexivity

The research team reflected racial and ethnic diversity (Black, white, and Indian) and gender diversity (female and male). The team also has differing experiences with immigration. The first author is an African-born immigrant, the second author does not identify as an immigrant, and the third author is a first-generation immigrant whose parents are from India. All authors have unique interest in studying and addressing the health inequities experiences affecting vulnerable population, particularly immigrants living in the USA. They consider themselves advocates for racial and ethnic data disaggregation and immigrant inclusion within public health research.

## Results

### Quantitative Findings

As shown in Table 1, 62% of all women reported current cervical cancer screening. The average age of all women was 37 (SD, 12.1) years. There was a significant age difference between women that reported current cervical cancer screening than those not current (39, SD 11.6 vs. 34, SD 12.5). Most women were unmarried (54%), had at least a high school education (81%) and less than $50,00 annual income (59%). Only 13% of all women did not have health insurance. The proportion of uninsured women that reported current cervical cancer screening was significantly different from those not current (9% vs. 19, *p* < 0.01). In terms of personal network characteristics, 23% of women reported high-social connectedness, 14% reported high-social support, 86% reported having health advice support, and 44% reported having high cancer screening support when needed. A greater proportion of women that reported current cervical cancer screening than those no current had high-social support (18% vs. 8%, *p* < 0.05).

**Table 1:** Descriptive and personal network characteristics of Black immigrant women

Personal characteristics	Total sample (*N* = 204) *n* (%)	Pap-test current (*n* = 127) *n* (%)	Pap-test not current (*n* = 77) *n* (%)	*p*-value
Cervical cancer screening				
Yes	127 (62.2)			
No	77 (37.8)			
Age (SD)	37 (12.1)	39 (11.6)	34 (12.5)	< 0.01
Married				0.077
Yes	93 (45.6)	64 (50.4)	48 (62.3)	
No	111(54.4)	63 (49.6)	29 (37.7)	
Education				0.126
No HS	25 (12.3)	16 (12.6)	9 (11.7)	
HS/some college	60 (23.4)	31 (24.4)	29 (37.7)	
College +	119 (58.3)	80 (63.0)	39 (50.6)	
Income				0.053
< 25 k	54 (26.5)	26 (20.5)	28 (36.4)	
25– < 49.999 K	67 (32.8)	45 (34.4)	22 (28.6)	
50–74.999 K	41 (20.1)	25 (19.7)	16 (20.8)	
75 k +	42 (20.6)	31 (24.4)	11 (14.3)	
Insured				
Private	94 (46.1)	72 (56.7)	22 (28.6)	0.003
Public/private and public	84 (41.2)	44 (34.6)	40 (51.9)	
No	26 (12.7)	11 (8.7)	15 (19.5)	
Health status				0.635
Excellent/very good	75 (37.5)	44 (35.2)	31 (41.3)	
Good	74 (37)	49 (39.2)	25 (33.3)	
Fair/poor	51 (25.5)	32 (25.6)	19 (25.3)	
Length of US residence				0.027
< = 5	31 (15.2)	13 (10.2)	18 (23.4)	
6–15	54 (26.5)	54 (26.0)	21 (27.3)	
16 +	119 (58.3)	119 (63.8)	38 (49.3)	
Social connectedness				0.077
High	47 (23.0)	35 (27.5)	12 (29.9)	
Medium	109 (53.4)	67 (52.8)	42 (54.5)	
Low	48 (23.5)	25 (19.7)	23 (15.6)	
Sociability score				0.831
High	11 (5.4)	6 (4.7)	5 (6.5)	
Medium	123 (60.3)	78 (61.4)	45 (58.4)	
Low	70 (34.3)	43 (33.9)	27 (35.1)	
Social support				0.038
High	29 (14.2)	23 (18.1)	6 (7.8)	
Medium	84 (41.2)	45 (35.4)	39 (50.6)	
Low	91 (44.6)	59 (46.5)	32 (41.6)	
Health advice support				0.764
Yes	168 (85.7)	103 (85.1)	65 (86.7)	
No	28 (14.3)	18 (14.9)	10 (13.3)	
Screening support				0.753
High	90 (44.1)	58 (45.7)	32 (41.5)	
Medium	69 (33.8)	43 (33.8)	26 (33.8)	
Low	45 (22.1)	26 (20.5)	19 (24.7)	

Table 2 presents regression models of the relationship between network characteristics and cervical cancer screening. For the unadjusted regression models, women with high-social connectedness were more likely to report current cervical cancer screening than those with low-social connectedness (OR: 2.68, CI: 1.12, 6.46). Additionally, women with private insurance were more likely to report screening than those without insurance (OR: 4.46, CI: 1.77, 11.26). The relationship between network characteristics and cervical cancer screening was further examined after controlling for insurance, health status, age, marital status, and income, whereas these relationships were completely mitigated, having private insurance compared with none was associated with screening (aOR: 3.06, CI: 1.04, 9.02).

**Table 2:** Association between personal network characteristics with cervical cancer screening

	Cervical cancer screening OR (95% CI)			
	Unadjusted models	*p*-value	Adjusted model	*p*-value
Social connectedness				
High	2.68 (1.12, 6.46)	0.04	1.86 (0.51, 6.79)	0.40
Medium	1.47 (0.73, 2.94)	0.71	1.33 (0.57, 3.13)	0.95
Low	Ref			
Sociability score				
High	0.75 (0.21, 2.76)	.61	–	
Medium	1.09 (0.59, 2.01)	.55	–	
Low	Ref			
Social support				
High	2.08 (0.76,5.71)	0.01	2.19 (0.54,8.88)	0.11
Medium	0.63 (0.34, 1.16)	0.70	0.53 (0.26, 1.08)	0.02
Low	Ref		Ref	
Health advice support				
Yes	0.88 (0.38, 2.04)	0.76	–	
No	Ref			
Screening support				
High	1.32 (0.63, 2.78)	0.53	–	
Medium	1.21 (0.56, 2.63)	0.88	–	
Low	Ref			
Insured				
Private	4.46 (1.77, 11.26)	0.0002	3.06 (1.04, 9.02)	0.01
Public/private and public	1.50 (0.61, 3.69)	0.29	1.19 (0.42, 3.33)	0.31
No	Ref		Ref	

Adjusted model controlled for age, married, education, income, health status

### Qualitative Findings

We identified four distinct themes connecting Black immigrant women’s personal networks with cancer related knowledge and capacity to access cancer screening services. The first and second themes respectively described how women and their personal networks had limited knowledge of cancer and related preventive measures. Theme three highlighted the vital roles of women’s social connections to their care seeking efforts. The fourth theme revealed that the influence of women’s personal networks on their cancer preventive behaviors were mixed. These themes provide contextual insights on the extent to which women’s personal networks facilitated screening behaviors like Pap test uptake.

#### Women Had Limited Cancer Knowledge and Related Prevention

Most women had little to no knowledge about different types of cancer and could not explain how cancer is caused, diagnosed, or prevented. Participants who had some knowledge about cancer identified the types that affects women. They mostly referenced cancers that directly impacted someone they knew. One woman mentioned, “Uhm I heard about the ovarian and yeah another one I also experienced was my stepmom…You see she passed away in 2016 and I uhm already forgot to even mention that she passed away with cancer…” (Participant 05, age 34). Another woman said, “Yes, there is uhm the breast cancer, the lungs one, the uterus one and then I, I know someone who had … what’s the name … oh my god, I forget the name.” (Participant 08, age 28). Besides mentioning the cancer types, the participants were unable to provide additional details about the cancer etiology.

Other study participants mentioned their lack of knowledge about cancer causes and prevention. One woman explained, “I don’t know exactly what causes the cancer, I don’t know if it’s in the blood or the vein or I don’t know, that’s what I don’t know how to say to prevent, to prevent the cancer” (Participant 09, age 63). Even women that mentioned that they had regular health checkups including cancer screening were unable to provide any information about cancer risks and prevention. One participant emphasized this point: “No, I don’t know anything about cancer, but I go for my own checkup every year” (Participant 06, age 56). Overall, when asked about cancer prevention most participants simply stated that they “don’t know.” The few women who described cancer prevention methods offered inaccurate information related to their folk remedies. Their responses included “drinking lemon water”, “avoid eating canned food”, and avoid using “soap or cream to get the light skin” to prevent cancer.

#### Personal Networks Had Limited Cancer Knowledge

Participants indicated that their close network members and members of their communities also generally had limited knowledge about cancer and related prevention. One noted that cancer prevention was not important to her friends in the USA and abroad, due to “lack of knowledge, lack of resources, lack of…ignorance is bliss, basically” (Participant 10, age 44). Another woman further described the extent of misinformation is often exchanged through her network and community members:

“Uhm, well I really don’t know much like I say sometimes I’m actually in some kind of [WhatsApp?] groups where we share information that would help us you know in our community and stuff like that so in this group people come, people post all kind of things about cancer and the things that you can do to prevent it like some herbal stuff, some natural stuff you know that are helpful… Like little things that you can do and use to prevent it” (Participant 05, age 34).

The woman’s comments further suggest that community members relied more on folk remedies and generational knowledge to fill the gaps of their limited cancer understanding. This colloquial information was more readily shared instead of medically validated information regarding cancer prevention like Pap tests.

One participant elaborated, “nobody talks about [cancer prevention and treatment]… they don’t like to, especially uhm the older, the generation before use they were very uhm, hm like a little skeptical about you know seeking medical help because they used to have a lot of herbal remedies” (Participant 10, age 44). Her statement echoes that sentiment of most participants who also perceived that their personal networks did not only have inadequate information about cancer but were not intentional about seeking accurate information about cancer control.

#### Social Connections and Health Seeking Behavior—General Network Role

Most participants identified 2–3 network members that provided support with health information and decision making. Co-workers, friends, and immediate family were all referenced as providing support for health-seeking behaviors, but mother figures were identified as the most common social tie resource. The influences of these network ties varied from casual check-ins to active participation in the woman’s health decisions. Notably, some participants believed their mom, stepmom, or mother-in-law played a more active role in their health decisions, and many felt more comfortable discussing their health decisions with these mother figures. On women commented, “[My mom is] the only one I have so, believe me every time she’s ready to you know be with me, anytime I go to the doctor I am with my mom, so I can tell you she’s uhm my everything, she’s the only one who give me uh advice if I don’t want to do something she’s the only one who tell me to do.” (Participant 06, age 56). Another participant explained how her mother encouraged preventative health behaviors. In this participant’s view her mother “heard about the cancer, the breast cancer, so she felt concerned, and she was just like she’s going to check all her kids to see if uhm is nothing wrong with them, yeah” (Participant 08, age 28). Other women described similar levels of concern expressed by their personal networks in ensuring that they access and received care for their general health related needs.

Close personal networks that provided support for women’s health-seeking behavior other than the participants’ mothers, usually had a more utilitarian role such as translation assistance or connecting them to a medical provider. For example, when asked about who they ask for medical help or advice, one woman (Participant 09, age 63) stated that her close friend, “…help[s] me because uh my English no good. She call, when I call [my co-worker] helping me to talk to the doctor or something like that, she’s uh help me, yeah.” Other women indicated that when they first migrate to the USA, they depended on their networks to help them navigate the healthcare system. One woman further explained, “Well, you know when you first come you don’t know anyone … you have to talk to friend’s which clinic do I go … so, I know I did that because I’m here 20 years…” (Participant 02). While this support was useful for the women, a few indicated that they tended to utilize their social networks less for health-seeking once they found medical providers or developed English-speaking fluency.

#### Cancer Specific Network Role

While most women identified network members that supported their general health-seeking behaviors, the members’ influence on their cancer-prevention behaviors varied greatly. About half of participants identified a network member that provided knowledge, advice, and tangible support which influenced cancer-specific screening, while the other half was emphatic about the absence of support from their personal networks in that regard.

Support received from women’s network members included encouragement to get cancer screening, transportation or assistance to get to a health facility for screening, and in some cases language translators during the physician–patient visits. For instance, one participant (Participant 06, age 56) said her mother, “knows the importance of having the [mammogram] exams… [and] asked me every time what’s the date you go for your mammogram stuff like that, yeah,” which motivated her to stay current on her breast cancer screening. Among participants who received cancer-specific health advice, their description of their interaction with their personal networks suggest that some of the network members had limited cancer knowledge. One participant (Participant 02) received the following cancer-prevention recommendation from a friend: “…don’t do too much sugar … they say that yeah, the cancer likes sugar too much so if you avoid the sugar it’s good, yeah because they say that.” Similarly, another participant (Participant 03, age 45) indicated she was told “that uh people shouldn’t drink too much [inaudible] or something like that. They say it’s to prevent cancer.” These women perceived a strong network support for cancer preventive behaviors, although they did not offer illustrious details of cancer prevention recommendations from their network member.

Other women did not receive cancer-specific advice or knowledge from their personal networks at all, and as one participant (Participant 09, age 63) explained, “I don’t have this chance [to talk about cancer prevention] uh nobody told me about this, I don’t have this chance, I don’t, nobody told me.” Because network members often had a gap in their cancer-specific health knowledge, about half of all participants stated that they relied on information shared from their medical providers to inform cancer-specific prevention and care. One participant (Participant 02) recalled, “ok my primary care doctor and gynecologists they always encourage women to do your mammogram anybody who turn 40 is supposed to do a mammogram every year and also every woman supposed to do pap smear every 2–3 years.”

### Integration of Quantitative and Qualitative Findings

The above themes offer a nuanced understanding of how social connectedness and social support may be related to cervical cancer screening uptake among the BIW. We observed a significant difference in the bivariable associate between social support and cervical cancer screening. Also, high-social connectedness increased the odds of cervical cancer screening in the unadjusted model. Exploration of BIW’s experiences with their social networks further suggests that these findings may be due to the various roles their network members played in their health-seeking behaviors including the promotion of cancer preventive behaviors. Comparing the quantitative and qualitative findings allowed us to observe the degree to which being connected to various members of their network is essential to the types of cancer prevention support that may be available to the women [54].

## Discussion

In this study, we found that women’s personal networks played a role in their uptake of cervical cancer screening. Women who reported high-social connectedness had higher odds of cervical cancer screening compared to those with low-social connectedness. After adjusting for demographic variables and health insurance, this association was attenuated. The qualitative findings provide additional insights into how the women’s network members support, encourage, and motivate them to engage in cancer screening behaviors including cervical cancer screening. Despite having limited knowledge about cancer preventive measures some women indicated that their close personal networks motivated them to seek cancer screening, helped them find a local clinic, and/or served as a translator within the medical setting.

Our study findings are similar to previous research that suggest that one’s network members can be influential in supporting cancer screening uptake including utilization of Pap screenings [38, 41, 69, 70]. It contributes to the limited quantitative research on the relationship between personal networks and cervical cancer screening. Earlier studies conducted by Suarez and colleagues indicated that high-social connectedness was associated with Pap screening among older Mexican–American women [71], and of four US Hispanic groups examined (Mexica, Cuban, Central, and Puerto Rican) the association was significant among Mexican–American women [38]. The relationship between social connectedness and cervical cancer screening may in part be explained by the supportive role of network members. In our study, we found a significant difference in the association between social support and screening, although, this relationship was mitigated after adjusting for other factors. To date, one other study found an independent association between social support, in the form of positive social interactions and greater affectionate support from social ties, and Pap screening among African immigrant women [69]. In addition to social support, other factors likely explain our finding of an attenuated relationship between social connectedness and cervical cancer screening after adjusting for our control variables.

For example, health insurance is an important predictor of cervical cancer screening. Adunlin and colleagues’ recent systematic review of personal and system level factors associated with breast and cervical cancer screening among immigrant women revealed that insurance coverage was an important predictor of cervical cancer screening [34]. The majority of the women in our study indicated that they had health insurance, which may explain the limited impact of social connectedness and support on cervical cancer screening uptake in our quantitative findings. Yet, it is worth noting that previous research suggests that immigrants from LMIC regions are more likely to be undocumented or have jobs that do not offer health benefits [72]. Also, as suggested in our qualitative findings the personal networks of Black immigrant women can be leveraged to facilitate support for cervical cancer screening. Therefore, it is important to consider ways to address access barriers to screening—e.g., affordable insurance coverage and free screening services—while developing program interventions to enhance the role of women’s personal networks to promote screening adherence.

Similar to participants in our study, immigrants from LMICs generally lean on their personal networks to help navigate the US health care system for both treatment and preventive services [34]. In the case of Black immigrants, they often contend with barriers including affordability, language, and cultural or religious differences [70]. Our qualitative findings suggest that despite having a small number of close network members, women’s network members contributed to addressing these barriers by connecting them to low-cost and/or culturally competent providers and serving as translators during healthcare visits when translation services were unavailable. We also found that many of these network members had inadequate knowledge about cancer and related preventive measures. Hence, additional research is needed to further understand the cancer education needs network members, as well as intervention strategies that will enable them to support Black immigrant women’s uptake of cervical cancer screening. This endeavor will address the call for cancer screening programs that leverage the social support networks of immigrants from LMIC regions [69, 73].

Future research should examine the role of close family network members, as evidence suggests that they are critical in informing health related decisions and providing support for health care access and use among immigrants [74]. For immigrant women, these family ties can provide different types of support for cancer screenings including pap testing [75]. Our study highlighted women’s perception of their mother-figures (e.g., mothers, mothers-in-law, and stepmom) as being essential to their cancer prevention behaviors because of the different types of support they provided. Thus, these network members should be meaningfully engaged in research to develop and disseminate culturally appropriate cancer screening interventions to reduce the risk of cancers among vulnerable populations like Black immigrant women. Interventions focusing on mother-daughter relationships specifically could be especially beneficial, since women felt more comfortable discussing health concerns with their mother figure. Prior work has shown evidence of the feasibility of using this dyad to promote cervical cancer prevention [76].

### Study Implications

Findings from the present study suggest numerous implications for improving access to cervical cancer screening uptake among Black immigrant women. First, promoting cervical cancer awareness and prevention among Black immigrant women should particularly focus on the women’s personal networks. These efforts should prioritize understanding the unique needs of women in terms of how they navigate the US healthcare system and utilize social networks to improve their access and use of preventative care. Second, longitudinal studies on Black immigrant women’s knowledge and awareness of cancer prevention and trends in their cancer preventive behaviors should be conducted. Such work can inform the implementation of tailored public health interventions among a Black subpopulation population with low cervical cancer screening rates. Third, policymakers should insider adopting new policies that promote culturally competent cancer prevention care, as these efforts would not only benefit the Black immigrant community, but other populations experiencing health disparities. Finally, additional health policies should engage key network members in developing and implementing community-based interventions to facilitate Black immigrant women’s adherence to screening behaviors.

### Limitations

The strength of this study is the use of a mixed-methods approach to examine the role of social connectedness in cancer screening among Black immigrant women. However, a few limitations of the study are worth noting. Egocentric network data is very useful in understanding interactions among network members but precludes the viewpoints of women’s network members. The quantitative data was not representative of Black immigrant women in the USA, as it was from a cross-sectional survey of a convenience sample of women. Though this approach allowed us to easily collect information from a hard to reach and unrepresented population, it can potentially introduce selection bias. Also, study participants may be limited in terms of having diverse backgrounds and perspectives on the research topic. The qualitative interviews were conducted by a white woman who was not part of the Black immigrant community. This may have resulted in response bias due to challenges in establishing rapport with various Black immigrant women. We worked closely with a key community leader from the community in recruiting and interacting with women, in attempt to address this concern.

## Conclusion

Network characteristics including social connectedness and social support are critical interpersonal level factors that promote cancer preventive behaviors. Despite low cervical cancer screening rates among Black immigrant women, there is growing evidence that leveraging social support can improve screening uptake. Our findings suggest that due to the supportive roles network members may provide women, it is necessary to explore the unique network dynamics of women. This approach may offer insights into how the role of different network members could evolve and potentially be enhanced to create opportunities for women’s involvement in health promoting behaviors such as early cancer detection, prevention, and treatment. Future research should investigate this relationship further to inform culturally competent public health interventions and clinical care that leverage the critical role of personal networks.
